# Health services reform in Bangladesh: hearing the views of health workers and their professional bodies

**DOI:** 10.1186/1472-6963-11-S2-S8

**Published:** 2011-12-21

**Authors:** Anne Cockcroft, Deborah Milne, Marietjie Oelofsen, Enamul Karim, Neil Andersson

**Affiliations:** 1 CIET Trust Botswana, PO Box 1240, Gaborone, Botswana; 2 CIET Trust, 71 Oxford Road, Saxonwold, Johannesburg, 2196, South Africa; 3 Institute for Democracy in South Africa (IDASA), PO Box 56950, Arcadia, South Africa; 4 HLSP Institute, SCH, 20 Upper Ground, London, UK; 5 Centro de Investigación de Enfermedades Tropicales, Universidad Autónoma de Guerrero, Acapulco, Mexico

## Abstract

**Background:**

In Bangladesh, widespread dissatisfaction with government health services did not improve during the Health and Population Sector Programme (HPSP) reforms from 1998-2003. A 2003 national household survey documented public and health service users' views and experience. Attitudes and behaviour of health workers are central to quality of health services. To investigate whether the views of health workers influenced the reforms, we surveyed local health workers and held evidence-based discussions with local service managers and professional bodies.

**Methods:**

Some 1866 government health workers in facilities serving the household survey clusters completed a questionnaire about their views, experience, and problems as workers. Field teams discussed the findings from the household and health workers' surveys with local health service managers in five *upazilas* (administrative sub-districts) and with the Bangladesh Medical Association (BMA) and Bangladesh Nurses Association (BNA).

**Results:**

Nearly one half of the health workers (45%) reported difficulties fulfilling their duties, especially doctors, women, and younger workers. They cited inadequate supplies and infrastructure, bad behaviour of patients, and administrative problems. Many, especially doctors (74%), considered they were badly treated as employees. Nearly all said lack of medicines in government facilities was due to inadequate supply, not improved during the HPSP. Two thirds of doctors and nurses complained of bad behaviour of patients. A quarter of respondents thought quality of service had improved as a result of the HPSP.

Local service managers and the BMA and BNA accepted patients had negative views and experiences, blaming inadequate resources, high patient loads, and patients' unrealistic expectations. They said doctors and nurses were demotivated by poor working conditions, unfair treatment, and lack of career progression; private and unqualified practitioners sought to please patients instead of giving medically appropriate care. The BMA considered it would be dangerous to attempt to train and register unqualified practitioners.

**Conclusions:**

The continuing dissatisfaction of health workers may have undermined the effectiveness of the HPSP. Presenting the views of the public and service users to health managers helped to focus discussions about quality of services. It is important to involve health workers in health services reforms.

## Background

Health care is a labour intensive enterprise in which knowledge, skills, and attitudes of health workers (in sufficient numbers) are crucial to providing a quality service [1,2]. A number of studies report seeking the views of different cadres of health workers about their work [3,4]. The attitudes and reactions of health workers might be pivotal to the success of health care reforms [5-9].

Bangladesh is a low-income country, with 81% of the population living on less than USD 2.00 per day. The country has poor health indicators, and important gender and other inequalities [10]. Several studies have reported public and health service user dissatisfaction with quality of service from government hospitals and other health facilities [11-15]. A number of donor-supported programmes have supported reform of health services in Bangladesh, especially for the poorest and most vulnerable in the population [16,17]. A prominent aim of all the reform programmes was to make health services more responsive to the needs of the population. They did not have improvement of conditions of service for health workers as a primary goal, but they did involve reorganisation of service delivery. The Health and Population Programme (HPSP) 1998-2003 [16] attempted to unify health and family planning services, previously run separately, right from the top echelons in the bureaucracy down to the primary care level. This led to dissatisfaction with the reform programme among health workers at all levels [5].

Most reported studies of health services quality, particularly in relation to services reform, focus either on the views and experience of service users, or on the attitudes and practices of health workers. Yet the two intertwine. A key aspect affecting satisfaction of health service users is the respect, or lack of it, from health workers [18]. Health workers who feel undervalued and mistreated by their employers are not in a good frame of mind to treat service users well.

We undertook three linked and nationally representative surveys of the public and health service users, to seek their views and experience about health services during the HPSP of 1998-2003. We describe the third national survey and its findings elsewhere [14,15]. In 2003 field teams interviewed respondents in a nationally representative sample of 25,490 households; 21,540 household members had recently used health services. Household opinions, use, and experience of government health services in 2003 were worse than in the surveys in 1999 and 2000. By 2003, only 10% of households rated government health services as “good”, and only 13% of health service users visited government facilities. Only 23% of government health service users got all the prescribed medicines from the facility, 55% got explanations of their treatment, 18% made direct payments to service providers, and 54% expressed satisfaction with the service. In 2003, we also undertook a survey to seek the views of health workers (predominantly in primary care settings) and discussed the findings from the public with local health managers and with national bodies representing health care professionals. This paper describes the views of the health workers, health managers, and professional bodies, and their reaction to the opinions and reported experience of the public and service users.

## Methods

The CIET international ethical review board approved the project, including the three household surveys and associated health worker surveys, before the first survey in 1999.

Alongside the 2003 household survey in Bangladesh [14,15], field teams visited government health facilities serving the sample sites and interviewed health workers present in these local facilities. The facilities included primary care facilities (union health and family welfare centres) and secondary care facilities - the *upazila* health complexes (UHC). Field teams left questionnaires at the UHC for self-administered completion. At union level the field teams administered the same questionnaire in face to face interviews with health workers. The anonymous questionnaire included questions on: personal characteristics; training and length of service; any difficulties faced in their work; specific questions about unavailability of medicines, unofficial payments, and behaviour of patients; and suggestions for improving their employment situation and the service to patients.

Based on preliminary analysis of the findings of the household survey, we developed a format to feedback and discuss key findings with local health managers, and professional associations. We identified five *upazilas*(administrative sub-districts) across the six divisions in the country where the users of government services were relatively satisfied with their service contact. In reality there was not much difference between these and poorly rated *upazilas*; no *upazila* rated well across all aspects of service experience. Members of the research team presented the findings about rating of services by the public and about experience and satisfaction of users to the *upazila* health and family planning teams, including doctors, nurses and paramedical workers. They invited the participants to discuss what they thought could improve satisfaction of services users, and the problems faced by health workers and how to improve their situation.

We discussed the findings of the public and health worker surveys with the Bangladesh Medical Association (BMA) executive and the Bangladesh Nurses Association (BNA) executive. They discussed the low satisfaction of patients with government health services, the issue of the unqualified practitioners many people used as their main source of health care, and the problems faced by doctors and nurses in their work.

### Analysis

Data operators entered the data from the local health workers' questionnaire using Epi Info, with double data entry to minimise key stroke errors. Analysis of quantitative data relied on CIET map open source software [19] which offers a user-friendly interface to the public domain R software. We examined associations between variables and outcomes in bivariate and then multivariate analysis based on the Mantel Haenszel procedure [20], using a step-down approach to final models of variables significantly associated with the outcome. We describe associations using the adjusted Odds Ratio (ORa) and the 95% confidence interval (CI).

Reporters took notes of the discussions with the health service managers in the *upazila* meetings and of the discussions with the BMA and BNA. We reviewed these reports to characterise themes in responses and extract relevant quotes.

## Results

### Views and experience of local health workers

We collected information from 1866 health workers at *upazila* and union level: 73% (1368) were paramedical workers, 17% (314) were nurses, and 184 (10%) were doctors. Overall, 50% (941) were male: 89% among doctors, 55% among paramedical workers, and 9% among nurses.

Nearly half of the respondents overall (45%, 833/1857) said they had difficulty fulfilling their duties. In a multivariate analysis including job, age, and sex, all three variables remained in the final model. Doctors were more likely to report difficulties (ORa 3.19, 95% CI 2.31-4.41), as were respondents under 40 years old (ORa 1.29, 95% CI 1.06-1.56); men were less likely to report difficulties (ORa 0.72, 95% CI 0.59-0.88). Table 1 summarises the difficulties cited by health workers in response to an open question. All three occupational groups mentioned inadequate supplies and poor infrastructure. Nearly a third of doctors and nurses and rather less paramedics (not all of whom had direct contact with patients) noted patients' behaviour as a problem. Nurses and paramedics rarely mentioned bad behaviour of colleagues, including corruption, but about one in six doctors mentioned this. Some paramedics (those who did outreach work) mentioned difficulty of access to some areas. Over a third of doctors cited administrative difficulties including: lack of clear policies, bureaucracy and complicated government rules, unclear demarcation of duties and responsibilities, difficulties in exercising authority and disciplining employees, and a gap between responsibility and authority. Respondents' suggestions for what would help them to work better largely mirrored the difficulties they had noted.

**Table 1:** Difficulties in fulfilling duties cited by local health workers

Difficulties	% (number)			
	Doctors n=182	Nurses n=309	Paramedics n=1356	Total n=1847
Inadequate supplies and logistics	33 (60)	54 (168)	37 (503)	40 (731)
Inadequate infrastructure / physical facilities	41 (75)	48 (148)	28 (384)	33 (607)
Lack of trained human resources	44 (80)	34 (105)	23 (307)	27 (492)
Patients' lack of understanding /bad behaviour	29 (53)	30 (92)	15 (196)	19 (341)
Areas difficult to access	6 (11)	1 (4)	23 (305)	17 (320)
Lack of incentives and personal benefits	10 (18)	6 (17)	19 (257)	16 (292)
Administrative problems	37 (67)	6 (17)	5 (69)	8 (153)
Bad behaviour of colleagues / corruption	16 (29)	8 (24)	3 (41)	5 (94)

Just over half the respondents (57%, 1066/1365) reported receiving fair treatment as employees. But only a quarter of doctors thought this (26%, 48/184). The main types of unfair treatment they cited were: poor salaries and benefits, lack of incentives for good work, and recognition (such as promotion) not being according to qualifications.

Nearly one half of the health workers (47%) said they had problems with the way patients behaved towards them: 66% of doctors, 64% of nurses, and 41% of paramedics. Nearly all said the problem was the “bad attitude” of patients, and two-thirds cited lack of medicines and patients blaming them for this, even accusing them of stealing and selling the government supplied drugs. Some 16% said they had problems with patients requesting benefits or services they were not entitled to – essentially a solicitation for corruption. Nearly half (46%) of doctors said this was a problem.

In the 2003 household survey, many patients complained about lack of medicines in government facilities and having to make unofficial payments to health workers [15]. Nearly all (94%) of the health workers blamed lack of medicines on inadequate supply to the facilities; only 5% cited corruption as a cause. A majority said unofficial payments were rare or denied they happened at all; few acknowledged these payments were common (Table 2). In response to an open question, the most common suggestions for how to stop unofficial payments were to increase monitoring and supervision (56%), to increase salaries (30%), and to improve health workers' morale and training (29%).

**Table 2:** Health workers' reports about the frequency of unofficial payments to health workers in government health facilities (among those who responded to the question)

Perceived frequency of unofficial payments to health workers	% (number)			
	Doctors n=180	Nurses n=292	Paramedics n=1343	Total n=1815
It does not happen at all	9 (17)	12 (34)	22 (291)	19 (342)
It is rare	64 (116)	71 (206)	56 (747)	59 (1069)
It happens sometimes	21 (37)	14 (40)	17 (225)	17 (302)
It is common	6 (10)	4 (12)	6 (80)	6 (102)

A quarter of respondents (25%) thought quality of work or service had improved as a result of the HPSP (Table 3). Overall, 18% thought nothing had changed; 24% of doctors thought this. The most notable specific change noted by respondents was unification or coordination of health and family planning services at local level. One out of six doctors (17%) complained of increased complexity of work and lack of clarity under the reforms.

**Table 3:** Changes as a result of the HPSP identified by health workers*

Changes identified	% (number)			
	Doctors n=162	Nurses n=265	Paramedics n=1313	Total n=1740
Quality of work or service improved	16 (26)	26 (68)	27 (349)	25 (443)
Unification/coordination of health and FP services	18 (29)	13 (33)	23 (300)	21 (362)
Nothing has changed	24 (38)	16 (43)	17 (228)	18 (309)
Changes, unspecified	12 (19)	32 (85)	7 (91)	11 (195)
Work more complicated/lack of clarity	17 (28)	5 (13)	8 (108)	9 (149)
Positive health impact	3 (5)	10 (26)	7 (86)	7 (117)
Increased patient awareness	3 (5)	3 (8)	8 (100)	7 (113)
Increased health role & decreased FP role	6 (10)	0 (0)	5 (62)	4 (72)
Increased work load and responsibilities	4 (6)	0 (0)	4 (46)	3 (52)
Services provided at grassroots level	3 (5)	0 (0)	3 (35)	3 (50)
Conflict between health and FP	5 (8)	0 (1)	3 (41)	3 (50)

*Respondents could give more than one response

FP=family planning

### Discussions with local service managers and professional bodies

#### Dissatisfaction and complaints of government health service users

Faced with the evidence that many patients were not satisfied with the behaviour of government health workers and more expressed satisfaction with private and unqualified practitioners, the *upazila* health teams suggested patients had unrealistic expectations of services from government facilities. They said staff shortages, poor conditions of work and low job satisfaction of staff, and lack of medicines and equipment all limited service delivery. Private practitioners, qualified and unqualified, treated their service as a business and therefore prioritised keeping their customers happy, even if what they offered was not “good medical practice”.

“They get cash, so they try to make patients happy by giving instant relief, for example intravenous saline. A qualified practitioner will never do this. Patients don't understand this.”. Member of an *upazila* health team

They said private practitioners had lower patient loads and were able to spend more time with each patient, listening to them and explaining the condition and the treatment. However, unqualified practitioners pandered to local beliefs and misconceptions, to keep patients happy.

“In most cases there is nothing true or factual in his explanations. He knows the local people's psychology and he tries to satisfy them with explanations they are going to believe and understand.”. Member of an *upazila* health team

“The explanations they give are not correct; they provide wrong explanations to please patients, just as a business technique.”. Member of an *upazila* health team

Some of the members of the *upazila* health teams believed that patients should *not* receive full explanations about their condition, and that some were not capable of understanding explanations about their condition.

“Patients want to know everything about their disease, but they should not be told about all diseases.”. Member of an *upazila* health team

“Sometimes a patient's level of understanding is low, so that even if he is told about the disease he will not understand.”. Member of an *upazila* health team

Suggestions to improve satisfaction of service users centred around increasing human resources and supply of medicines. Many discussants were in favour of introducing official user charges, mostly because they thought these would decrease the patient load, although some also mentioned the money could be used to improve facilities.

“User charges will discourage the healthy people who visit the UHC unnecessarily, just to get medicines or just to pass the time.”. Member of an *upazila* health team

The executive of the BMA accepted that many patients were not satisfied with government health workers. They pointed out that the private practitioners, whose behaviour patients were more satisfied with, were the *same* doctors who worked in government facilities, doing private practice in addition to their government work. Thus these doctors had the skills to talk to patients, but could not do so in government facilities because of the poor working conditions and high patient loads. One member suggested that it was only literate patients who really expected or needed an explanation about their condition; most patients just needed treatment with sympathy.

“A man who arranges a marriage for his daughter does not feel the need to explain anything to her. What is needed for patients is to treat them with sympathy.”. BMA Executive Member

Members of the executive of the BNA argued that high patient loads led to the poor quality of interaction with patients in government health facilities.

“You can't talk to 60 patients so that you satisfy them, as you would be able to with 10 patients.”. BNA Executive Member

The BNA executive members recommended more in-service continuing education for nurses, including training about interaction with patients. Some stressed that health workers should be accountable to the population they served, as well as upwards through their management system; this could be encouraged through local bodies which included patient representatives.

#### Increasing use of unqualified practitioners

The executives of the BMA and BNA agreed with evidence from the household survey that many people used unqualified medical practitioners as their source of health care and the trend was increasing. The BNA recognised that if the government could not deliver a service, then the public, especially the poor, had no choice but to turn to unqualified providers. They felt unqualified practitioners should be phased out but as an interim measure they should receive training (from qualified nurses and doctors) to allow them to practise safely; they should be regulated and have clear guidelines on when to refer patients to qualified practitioners. The BMA Executive, in contrast, was strongly against the idea of providing training for unqualified practitioners, as this could encourage them to take on more responsibilities without understanding what they were doing.

“Don't try to qualify the unqualified.”. BMA executive member

The BMA said the government should increase the health budget and hire more qualified doctors at local levels, filling vacant posts.

#### The situation of health workers

Health service managers on *upazila* health teams identified with the problems and complaints voiced by the local health workers in the survey, particularly lack of staff, lack of resources (especially medicines) and lack of training opportunities. Managers posted staff to *upazilas* far from their homes, the housing was inadequate, there were problems of personal security, there were no good schools for their children, and there were no overtime payments or opportunities for promotion. Perks (such as training or meetings abroad) and promotion were not given according to merit, and favouritism was sometimes politically motivated. All of this led to low job satisfaction, low morale and no incentive to improve service delivery. These problems were still keenly felt despite the reforms of the HPSP.

The BMA executive expressed pleasure that the social audit sought views of doctors and other health workers, although they said this should have happened before beginning the HPSP. They said most problems with doctor-patient interactions were due to the poor working conditions of the doctors.

“A doctor in Dhaka medical college with good facilities for investigating and treating patients, and good personal conditions including accommodation, is able to provide a good service. The same doctor in a poorly equipped, understaffed UHC is not able to provide a good service to patients.” BMA Executive Member.

The BNA executive said nurses experienced very poor conditions of service, even in Dhaka. Security concerns for the female nurses were serious, especially in more remote *upazilas*. Nurses had no effective career progression, and felt frustrated by their exclusion from local planning and lack of decision-making latitude.

## Discussion

The concerns voiced by the health workers about their conditions of work and the problems they faced echo those reported in other studies from Asia and Africa [3,5,21-23]. Complaints about lack of staff and resources, poor conditions of work, and unfair treatment as employees seem to be nearly universal. The health workers in our survey in Bangladesh cited complaints from patients and bad behaviour from patients or their relatives as important among the problems they faced as health workers; this has not been emphasized in other reports. Overall, the complaints of the three categories of health workers were quite similar, but doctors stood out as being more likely to cite administrative problems, more likely to say that patients asked for services they were not entitled to, and more likely to feel they were unfairly treated as employees. Doctors, with higher qualifications and seniority, indeed have more administrative responsibilities than the other two staff groups. Most users of government health services in Bangladesh who reported unofficial payments said they paid the doctor [14], so it is not surprising that doctors as a group more often reported solicitations to corruption. The greater proportion of doctors feeling unfairly treated as employees may relate to higher career expectations, given their training and qualifications.

Few of the health workers we interviewed admitted to unofficial payments being common, even though one in five users of government health services reported unofficial payments to service workers. The interviewers did not ask the health workers directly if they had ever been offered or accepted an unofficial payment, but both health workers and patients may decline to talk about the topic at all [24,25]. Unofficial payments are an important source of dissatisfaction for users of government health services in Bangladesh and many other countries [24,26-29]. A study in Tanzania reported that unofficial payments were also related to lower job satisfaction and demotivation of health workers [30].

A minority (25%) of the health workers in the survey considered quality of their work or service had improved as a result of the HPSP and almost as many, particularly doctors, reported that nothing had changed under the reforms. Some doctors complained of complexity and lack of clarity and it was notable that the health workers did not report improved conditions of work as a result of the HPSP. The reform programme included an element intended to improve human resources management, which could have led to improved working conditions [31]. An important element of the HPSP was unification of the previously separate health and family planning services, leading to changing roles and management arrangements for many workers. The unification only ever took place to any extent at local level and reversed entirely with a change of government in 2001 [31]. The reforms also attempted to change the management culture by separating management from service provision; either skills development for doctors and nurses acting as managers or engagement of a separate cadre of managers intended to allow health professionals more time for direct service delivery.

Presenting health workers with evidence of what the public and patients think and experience was a useful way to focus the discussion on the problems and criticisms raised by service users and made it difficult for them to deny problems, but the approach could be confrontational if not handled with care. Even in carefully managed discussions, there was a tendency for the health workers and their professional organisations to blame others for problems: the patients for being uneducated, uninformed, and unrealistically demanding; or the government management of the services, for example for failing to deliver necessary medicines to *upazila* health facilities. Similarly, their potential solutions mostly held others responsible for solving problems: user fees would mean less patients used the services unnecessarily; a better system for providing medicines to *upazilas* would mean patients were more satisfied; and funds to increase medical staffing would mean doctors had time to treat patients better.

*Upazila* health teams and the health professional bodies welcomed involvement in the social audit. A specific complaint from the BMA executive committee in 2003 was implementation of the health reforms of the HPSP without consulting them. Other authors have noted that health reforms are unlikely to be successful without health workers' involvement at all stages of planning and implementation. [6,7] The complaint of the BMA about lack of consultation was despite the initial “highly participatory preparation approach” for the HPSP, which included 17 working groups of “key stakeholders” including members of the BMA and BNA to prepare different aspects of the programme [31]. All the technical task forces for HPSP formulation had members from the BMA and BNA. Both the BMA and BNA are politically divided, and their support for government led programmes depends on which fraction is in the executive committee at the time.

A major finding from the household survey was that patients are unhappy with the way health workers in government facilities behave towards them. The behaviour of health workers towards them is one of the main determinants of satisfaction of government health service users. This was clear in the household survey [15] and in surveys of patients in different areas in Bangladesh [11,13]. Faced with the evidence about poor interaction with patients, both the BMA and the BNA blamed working conditions, and down played any need to change doctors' or nurses' attitudes towards patients. However, there is evidence from an observer study that nurses in government hospitals in Bangladesh spend little time in direct patient contact [32], and qualitative evidence suggesting this is partly due to nurses avoiding patient contact because of cultural stigma attached to such contact [33].

A feature of health services use in Bangladesh is the continuing move away from government services towards private practitioners, and particularly unqualified practitioners (so-called “village doctors” or “quacks”). The use of unqualified practitioners increased over the period of the HPSP [15] and the reality is that government health services in Bangladesh provide very little of the basic health care in the country. A study in 2007 found that about two-thirds of care-seekers consulted village doctors first [34]. This preference for seeking health care from non-government providers is not confined to Bangladesh [25,35-38] and there is ongoing debate about the role of the private sector in health care in developing countries [39,40]. The very high use of unqualified practitioners of Western medicine is striking in Bangladesh. However, the BMA rejected all ideas of providing training or regulation of the non-medically qualified practitioners. This negative view from the body representing the medical profession in 2003 is relevant for initiatives to train and certify unqualified practitioners to help solve the problem of medical staffing in Bangladesh [34]. There are reports of successfully training village doctors in Bangladesh to diagnose and supervise treatment of tuberculosis [41]. The government engages around 70,000 non-medically qualified health workers of different types, with training, ranging from 40 days to six months, provided in the public and private sectors. Private institutions providing such training need to involve qualified doctors as a condition for accreditation by the government Health Directorate.

## Conclusions

The survey of local health workers provided evidence about their experience and problems, to set next to the views and experience of government health service users and non-users. Their negative views and continuing complaints went some way towards explaining why the health reforms did not improve experience of service users. Presenting evidence from the public to local service managers and professional bodies helped to focus discussions with them. Future health reforms will need to engage with health workers fully throughout the process to increase their chances of success.

## List of abbreviations used

BMA: Bangladesh Medical Association; BNA: Bangladesh Nursing Association; CI: Confidence Interval; HPSP: Health and Population Sector Programme; ORa: Adjusted Odds Ratio; UHC: *Upazila* Health Complex

## Competing interests

The authors declare they have no competing interests.

## Authors' contributions

AC helped to design and implement the research, undertook the analysis, and drafted the paper. DM helped to design and implement the research and contributed to drafting the paper. MO helped to design and implement the research and critically reviewed the paper. EK provided advice during the design and implementation of the research and contributed to drafting the paper. NA was responsible for the design, supported the implementation and analysis, and contributed to drafting the paper.
